# Open Cardiac Surgery during Profound Hypothermia

**Published:** 1962-01

**Authors:** A. J. G. Dickens


### OPEN CARDIAC SURGERY DURING PROFOUND HYPOTHERMIA

*A report of an early operation in Bristol*

BY

A. J. G. DICKENS

*Medical Student*

On Tuesday, 31st January 1961, I was privileged to assist at an operation performed by Mr. Ronald Belsey at the Bristol Royal Infirmary, for closure of an atrial septal defect (A.S.D.) under profound hypothermia. This was the first case to be allotted to a student dresser, although the sixth one in the profound hypothermia series in Bristol, and an account of it may be of both topical and historical interest.

The method was pioneered by Professor C. E. Drew, at the Westminster Hospital and the first reports were published in 1959 (Drew *et al.* 1959a and b). Since then the Westminster's series stands at over 110 cases, the largest total in the world using this method. The most important advance in it is that an oxygenator is no longer necessary, the patient's own lungs being used for this purpose. Two pumps, simulating the ventricles, maintain a double extra-corporeal circulation once ventricular fibrillation occurs at 30°C or below. The operation itself is performed at 13–15°C, and a state of suspended animation of at least one hour is safe at this temperature, although in Bristol 45 minutes is at present taken as the normal working limit. A less severe routine can be used in patients with the *secundum* type of defect, as this is now closed in 20 minutes at 17–20°C.

At present in Bristol the Intra-Cardiac Surgical Unit operates on one patient every Tuesday, and the patients are clerked by students from the Professorial Surgical Unit. The patients have beforehand been investigated either at the General Hospital or at Frenchay Hospital, and post-operative convalescence is often arranged at Frenchay or Bournemouth.

### THE APPARATUS

The apparatus consists of two pumps to replace both the ventricles, so set as just to occlude the resistance of a 100 cm. column of normal saline, and two reservoirs with translucent walls placed about 50 cm. below heart level. The blood from the atria enters these, and they are primed with 400–500 ml. of fresh heparinized blood. The right cardiac bypass circuit comprises reservoir and pump, and the left circuit reservoir, pump, and heat exchanger. The latter as used in Bristol consists of two to eight stainless steel tubes through which the blood passes in parallel, with Y-shaped connections dividing the entering blood stream into equal parts, and returning them to one stream after passage through the exchanger. The weight of the patient decides the number of tubes that will be employed, and they are then suspended in a trough so that warm or cold water can be run over them and so alter the blood temperature. Although this is not the most modern form of exchanger, it has the considerable merits of simplicity, even division of blood flow, low cost, ease of maintenance, and adaptability of size to the weight of the patient. There are four cannulae, one for each atrium, one for the femoral and one for the pulmonary artery; they consist of short straight thin-walled steel tubes, the atrial ones having fenestrated endings. The circuit components are joined by $\frac{3}{8}$ in. internal diameter polyvinyl chloride tubing, and priming of the whole apparatus pre-operatively needs 1 to 2·5 litres of blood, depending on the weight and size of the patient.

### THE METHOD

At operation, the patient is first heparinized, using 2 mg. per kilo. of body weight. The femoral arteries are exposed and cannulated, the left one with the return cannula from the left bypass, and the right one with a catheter for continuous recording of blood pressure. Median sternotomy is performed, bleeding points dealt with, and the pericardium is opened. In establishing the bypasses, the left circuit is first brought into action, and when this is working satisfactorily the right bypass is begun. The left atrial cannula is inserted, usually into the appendage, the pump is started, and the bypass comes into action.

The initial flow rate is 1 litre per minute, and this is gradually increased as the temperature falls. The same volume as drains into the reservoir is pumped from it through the heat exchanger and back into the femoral artery, and cooling is begun. Initially 25 per cent of the left atrial flow is passed through the pumps and heat exchanger, and this proportion is increased very slowly until the exchange transfusion has been completed. Now the right atrial cannula is placed in position, and as soon as ventricular fibrillation begins (at an oesophageal temperature of 27–29°C) blood is allowed to drain through it into the reservoir on the right bypass circuit. The pulmonary artery cannula is immediately placed in position through the right ventricular infundibulum and pushed through the pulmonary valve. The flow rate is increased in both pumps, and the left one is adjusted to give a mean B.P. of 70–80 mm. Hg in the systemic circulation. This determines the volume of blood returning to the right atrial reservoir, and the right pump is set to keep this level constant. Once stabilization has been reached, only minor adjustments are usually necessary. The flow rates normally aimed at are from 50–100 ml. per kilo per minute, being higher for a child than for an adult. When the patient's temperature has fallen to 13–15°C the pumps are stopped, the venae cavae occluded, and the aorta and pulmonary artery clamped off to prevent air embolism on cardiotomy. The heart is drained via the venous lines, and the actual surgical operation on the heart can begin.

After the operation, air is displaced from the heart by a few manual turns of the pump after removal of the appropriate great vessel clamps. When heart repair is complete the remaining clamps are released, the pumps are restarted, and warming commences. The heart may revert to normal sinus rhythm spontaneously at 26°C or upwards, especially in a child; or it may continue to fibrillate, and require electrical defibrillation at 34–35°C, when no further low temperature risks will be encountered. Re-warming is continued to at least 34°C, and then the pumps are slowed if the heart beat is satisfactory. The right bypass is stopped, and the cannulae removed, and then the left side is similarly treated. The ligatures are left untied as the cannulae are taken out to allow re-cannulation if necessary, and they are finally tied when the heart action is satisfactory. The B.P. catheter is withdrawn and the vessels repaired. Haemostasis is thoroughly ensured, and the pericardium and chest closed. The drainage tubes are inserted, one to the pericardial cavity and one to the anterior mediastinum; and the whole procedure is complete.

### SOME FURTHER OBSERVATIONS

Right ventricular bypass is usually postponed until the heart begins to fail at about 30°C, so that only two cannulae need attention at any one time, and there is sufficient time-margin for the correction of any difficulty that may be encountered. During bi-ventricular bypass the pumps may be working at different speeds, and some heart conditions may require that one or other pump should give a higher output, e.g. Fallot's tetralogy or a patent ductus arteriosus necessitate a higher left output, and severe left-to-right shunts or tricuspid or pulmonary regurgitation require a higher right output. Discrepancies in the flow of the two pumps have never yet caused any serious concern.

The actual flow rates used are higher than would be sufficient to satisfy body metabolism at lower temperatures, but heat exchange is made much quicker and easier by the flow being raised above the minimum for metabolic needs. The "core" organs of the body—brain, lung, liver, and kidney—cool and re-warm more quickly than the muscle mass, and care must be taken to avoid too rapid a change in temperature in these organs. Perfusion of a brain at body temperature with very cold blood, and perfusion of a cold brain with blood above normal body temperature may cause damage; and in Bristol these risks are always avoided by taking 45–50 minutes for cooling or warming the patient. During re-warming, warm water is also passed through a water-blanket under the patient so as to maintain body temperature while the chest is being closed.

The advantages of the operative method are that the surgeon works on an empty motionless heart produced by cooling alone, without the risks that attend the use of anoxia or drugs to produce this effect. There is no sinus flow, as there is with a normothermic complete cardio-pulmonary bypass. The E.E.G. is constantly monitored, as this gives early warning of an inadequate flow rate; so does the recording of the central arterial pressure. As mentioned above, slow cooling and warming reduce risks; in the case to be described they took $1\frac{1}{2}$ to 2 hours each, but in more recent operations the times have been reduced, as has the total length of the operation (to about six hours instead of ten).

### CASE HISTORY

The patient operated upon on 31st January 1961 was a 30-year old chef from Newton Abbot who is married with three children. He had always been told that he had "heart trouble", attending an open-air school when he was 8 years old. He had diphtheria and pleurisy before he was nine, but never rheumatic fever. His general health on admission to Bristol General Hospital on 23rd October 1960 was good, with a history of some left-sided chest pains over the previous two years, but these had not been severe until recently, and it was for these that he was admitted to Newton Abbot Hospital, whence he was transferred to Bristol under the care of Dr. D. H. Davies. He had never had any shortness of breath, or any difficulty in coping with his work, and at first his pain was thought to be that of a cardiac neurosis, or left mammary pain. However, a diagnosis of A.S.D. was made after investigation, and as this condition if untreated has a life expectancy of only 35 years or so, surgical treatment was obviously very desirable in this case.

Various features went towards making the diagnosis. On auscultation there was a pulmonary systolic murmur, and the second sound was split, independently of respiration. These were well shown by a pulmonary arterial phonocardiogram, which records four things: the heart sounds in the pulmonary area, the heart sounds in the third left intercostal space, the E.C.G., and the respiratory movements with their timing. The traces are on a paper marked in time intervals, and it could easily be seen that there was a pulmonary systolic murmur due to the increased flow, and that the second heart sound was always split by 0·06 seconds. Normally the second heart sound's components A2 and P2 are separated in inspiration only, and are together in expiration, but in this case they were separated in expiration also. The explanation is that normally right ventricular systole is more prolonged than left, so that the pulmonary valve closes after the aortic, and P2 is after A2. This is accentuated in inspiration when right ventricular filling is increased by low intra-thoracic pressures. In an A.S.D. there is a constant stream of blood passing through it, and when the right atrium fills in inspiration less blood comes through the defect but more comes in from the veins, so that the filling of the right ventricle is constantly maintained at an increased level and the second sound has a constant split. In expiration more blood comes through the defect. The E.C.G. confirmed the expected right ventricular hypertrophy.

A straight X-ray of chest showed characteristic features also. There was no rounding of the apex, as in left ventricular hypertrophy. The main pulmonary artery was more pronounced than that of the aorta, and the pulmonary arteries were well filled. There was a concentration of vessels adjoining the superior vena cava, with anomalous vessels draining this region. The diagnostic indications were the right-sided hypertrophy, small aorta, over-filled lung fields, and anomalous drainage on the right side. A tomogram series showed the pulmonary vessels very well, with the left lung draining to the left atrium, and also the right lower lobe, but the veins from the right upper and middle lobes were draining to the superior vena cava. A cardiac catheter could be passed from the right arm to the right atrium and thence into the coronary sinus, and up a persistent *left* superior vena cava to the left arm. When it was withdrawn to the superior vena cava proper it entered one of the anomalous veins leading from the cava. The pulmonary arterial pressure was normal, and the blood in the right atrium was more oxygenated than in both venae cavae, proving a left-to-right shunt. Thus the diagnosis was confirmed of an A.S.D. with anomalous pulmonary veins—a type that has been named a "sinus venosus" type of high A.S.D. Surgical treatment will cure this condition, but when closing the defect it is vitally important to ensure that the anomalous vessels are in fact diverted into the left atrium. If possible the lower margin of the defect is sutured to the wall of the superior vena cava above both anomalous veins, so redirecting them into the left atrium; the highest vein may even need re-implanting in the left atrial wall, or ligation if it is too high for the septum to be sewn above it.

### THE OPERATION

In this case Mr. Belsey decided to operate in January 1961 at the B.R.I. The patient's remaining teeth, which were in poor condition, were removed, and he was sent home for Christmas and re-admitted to the Intra-cardiac Surgical Unit on 21st January 1961. Pre-operatively his B.P. was 140/75, his Hb was 92 per cent, his prothrombin index 100 per cent and his electrolytes normal.

The day before operation, 20 pints of blood had been specially donated by Regional Blood Transfusion volunteers; this blood was citrated, so that any unused bottles could be returned to the Blood Bank, which would have been impossible with heparinized blood.

At the start of the operation, the patient, who weighed 70 Kg., was given 140 mg. of heparin, and after this 25 mg. of heparin were added just before use to each pint of added blood, of which there were 7, the remaining 13 being used to prime the apparatus. During the operation calcium gluconate was given at the rate of 5 ml. per pint of blood to counteract the cumulative effect of the citrate—the last 18 pints were so treated. Sodium bicarbonate was also given at the rate of 4 mEq. per Kg. This was to increase the alkali reserve, since some metabolism continues in the muscles even when none occurs in the "core" organs, and the bicarbonate was to counteract the metabolites. (In recent cases no bicarbonate has been given because the pH monitoring apparatus now used gives adequate warning of acidosis. Carbon dioxide is even used now, to maintain the cerebral vasodilatation that is very valuable in preventing cerebral damage).

Continuous records were taken of (1) the temperature of the water in the heat exchanger, (2) the amount and time of blood added, (3) the B.P. of the patient, (4) the E.C.G. and E.E.G. of the patient, (5) the flow rate of the two pumps, (6) the total fluid balance in and out, and (7) the temperature of the patient. This last was measured in two places, the nasopharynx (equivalent to brain temperature) and the oesophagus (equivalent to heart temperature); and readings were made in pairs in that order. Polybrene was used at the end of the operation as an antidote to the heparin—2 mg. per kilo in the first, and 1 mg. per kilo in the second dose.

A timetable of events is the easiest way to present an account of the operation.

| | |
|---|---|
| 8.00 a.m. | Premedication: 50 mg. pethidine, 25 mg. phenergan, and 1/150 gr. atropine. |
| 9.17 a.m. | Anaesthesia: 200 mg. thiopentone. |
| 9.20 a.m. | Curare, 60 mg. Nitrous oxide and oxygen. |
| 9.46 a.m. | Curare, 15 mg. |
| 10.20 a.m. | E.E.G., E.C.G. thermocouples in place. Femoral artery incisions. |
| 10.40 a.m. | Chest incised. Temps. 35°C. 35·8°C. B.P. 110/70. |
| 11.00 a.m. | Sternotomy and chest open. The marrow surface was coated with wax to stop haemorrhage and the periosteal surfaces were diathermized, but some oozing continued throughout the operation. |
| 11.11. a.m. | Heparin 140 mg. |
| 11.35 a.m. | B.P. catheter in R.femoral artery. B.P. 70. |

| | |
|---|---|
| 11.39 a.m. | L.femoral catheter in. |
| 11.45 a.m. | Pericardiotomy. |
| | The cavity contained some frothy fluid, due to a congenital defect in the left side of the pericardium itself, so that the cavity communicated with the superior mediastinum and the L.pleural cavity. This defect was not sewn up as the procedure would have been time-consuming. There was evidence of a past myo- and peri-carditis, causing adhesions between the two pericardial layers. |
| 12.15 p.m. | 540 ml. sodium bicarbonate infusion commenced. 15 mg. curare. |
| 12.37 p.m. | "L" atrial cannula in. (Actually into the R.atrium in this case, because the L.auricular appendage was small, and L.atrial drainage was easier at this stage via the A.S.D. with the cannula in the R.atrium; after correction of the defect, the cannula was transferred to the L.atrium before perfusion was re-started.) |
| 12.39 p.m. | On L.cardiac bypass. L.pump flow rate (L.F.R.) 1000 ml./min. |
| 12.44 pm.. | R.atrial cannula in. |
| 12.46 p.m. | Cooling commenced. L.F.R. 1500 ml./min. |
| | Temps. 34·4°C, 35·1°C. |
| 1.04 p.m. | Temps. 33·2°C, 30·2°C. B.P. 80. |
| | L.F.R. 3200 ml./min. |
| 1.07 p.m. | Auricular fibrillation. |
| 1.08 p.m. | Ventricular fibrillation. |
| 1.09 p.m. | Pulmonary artery cannula in. L.F.R. 3600 ml./min. |
| 1.10 p.m. | On R.cardiac bypass. L.F.R. 2000 ml./min. |
| 1.30 p.m. | Temps. 25°C, 21·6°C. B.P. 85. |
| | L.F.R. 3500, R.F.R. 2800 ml./min. |
| 1.57 p.m. | $N_2O$ off. 95 per cent $O_2$ and 5 per cent $CO_2$ given till 4.27 p.m. |
| 2.10 p.m. | Temps. 16·4°C, 15·2°C. |
| 2.18 p.m. | Temps. 15°C, 14°C. B.P. 75. |
| 2.19 p.m. | Pumps off. Inflation stopped. Heart drained. (From 1.10 p.m. the great vessels had been cleaned in spite of the pericardial adhesions, and tapes had been placed round the aorta, pulmonary artery, and superior and inferior venae cavae. These were occluded when the pump had been turned off, and then the heart was drained). |
| 2.24 p.m. | Temps. 13·8°C, 13·6°C.—the minimum reached. |

*Cardiotomy.* At 2.40 p.m. the R.atrium was opened and the defect was inspected. It was a large A.S.D. of the sinus venosus type, measuring 2 in. × 1½ in. The superior R.pulmonary vein entered the base of the superior vena cava, and a more inferior pulmonary vein orifice straddled the region of the posterior margin of the septum. The septal defect was closed in two layers with continuous 3/0 black silk sutures. The first suture was inserted in such a way that by taking multiple bites of the base of the superior vena cava and the wall of the atrium the orifice of the superior vena cava was separated from that of the superior pulmonary vein, and the latter vessel was diverted very satisfactorily into the L.atrium. As the last suture was put in, the L.atrium was filled to overflowing with blood to prevent any air embolism; and the last suture was drawn tight to close the defect actually below the surface of the blood welling up from the L.atrium through the defect. Any remaining air in the R.atrium would be picked up in the lungs and cause no harm. After this the atriotomy incision was closed with a double row of continuous 2/0 black silk sutures. The L.atrial cannula was now inserted into the L.atrial appendage.

| | |
|---|---|
| 3.10 p.m. | Temps. 14·6°C, 14·3°C. |
| 3.11 p.m. | Pumps on. Inflation re-commenced. |
| | L.F.R. 3000, R.F.R. 2000 ml./min. B.P. 45. |
| 3.30 p.m. | Warming commenced. Temps, 18·5, 18·5°C. |
| 4.00 p.m. | Temps. 22·4, 23·2°C. B.P. 55. |
| | L.F.R. 3700, R.F.R. 3200 ml./min. |
| 4.06 pm.. | Efforts at spontaneous respirations started. |
| 4.24 p.m. | Temps. 26°C, 29°C. B.P. 65. E.C.G. fibrillating. |
| 4.27 p.m. | $N_2O$ on again. |
| 4.36 p.m. | E.E.G. activity resuming. |

| | |
|---|---|
| 4.55 p.m. | Electrical defibrillation, now there was no further risk from the lowness of the temperature. Normal sinus rhythm resumed and maintained with just one shock. Temps. 31·4°C, 34·4°C. B.P. 70. L.F.R. 3200, R.F.R. 2800 ml./min. |
| 5.00 p.m. | $N_2O$ off, for last time. |
| 5.20 p.m. | Off right cardiac bypass. Right atrial and pulmonary artery cannulae out. Temps. 33·6°C, 36·6°C. B.P. 65. L.F.R. 2700 ml./min. |
| 5.30 p.m. | 300 ml. sodium bicarbonate infusion commenced. |
| 5.40 p.m. | Off left cardiac bypass. Left atrial cannula out. Temps. 35·8°C. 37·7°C. B.P. 60. |
| 5.50 pm.. | Polybrene, 140 mg. (All oozing promptly stopped). |
| 6.00 p.m. | Both femoral cannulae out (B.P. one ex-right femoral artery). Temps. 36°C, 36·1°C. B.P. 60. |
| 6.10 p.m. | Polybrene, 70 mg. |
| 6.30 p.m. | Two drainage tubes in place, one pericardial, one mediastinal. |
| 6.56 p.m. | Chest and femoral incisions closed with wire sutures. |
| 7.00 pm.. | Operation completed. Temps. 35·4°C, 35·8°C. B.P. 110/65. |

The heart in this case was stopped for 52 minutes, the longest up to then in the series, the previous times had been 30, 45, 39, 40 and 48 minutes. Cooling down took 1 hour 33 minutes, and warming up took 2 hours 10 minutes.

### POST-OPERATIVE COURSE

On return to the ward, the patient was grunting loudly, and respiration was laboured. Chest movement was poor, but better on the left, so an X-ray was taken which showed a right haemopneumothorax. Oxygen was given, and this kept the patient a good colour. He had a fair night, and at 9.30 a.m. next day Mr. Belsey put in a right intercostal drainage catheter; and this was followed by marked improvement. A later X-ray showed both lungs to be inflated. The pericardial and mediastinal drainage tubes were removed at 9.30 a.m. on 2nd February 1961, and the intercostal catheter at 5.0 p.m. that day. One pint of blood was infused as the haemoglobin level had fallen a little. The patient's electrolytes were normal, except for non-protein nitrogen 90 mg./100 ml. and chlorides 83 mEq./L. There was some surgical emphysema of the neck, but this gradually cleared without trouble.

On 4th February 1961 there were still some signs of left lung collapse, but these were improving. The patient was transferred to Frenchay on this day, with a drug therapy of digoxin 0·25 mg. b.d., penicillin 0·5 M units t.d.s., and streptomycin 0·5 gm. b.d. At Frenchay he had some right leg venous thrombosis which delayed his recovery for a short time, but he was sent home on 20th February 1961 feeling quite fit, although still getting tired easily. He is seen regularly at the monthly follow-up clinic for post-operative patients run jointly by Mr. Belsey and Dr. Davies, and he is attending at increasing intervals latterly, since he is back at work with no remaining disability at all, and with his heart size returned to normal.

*Acknowledgements*

I am very grateful to Mr. Belsey for his interest and encouragement with this paper and for his permission to write up the case with a description of the method and actual operation. Dr. D. W. Barritt gave much valuable help with the reasoning behind the diagnosis, and I should like to thank both him and Dr. D. C. Gengos for their assistance with this article on a subject that has ever-increasing applications in the field of modern surgery.

An account of this operation was presented to the Galenicals Society, University of Bristol, and was reported in *The Black Bag*, Vol. 18. No. 1.

### REFERENCES

Drew, C. E., and Anderson, I. M. (1959a). *Lancet i*, 748.
Drew, C. E., Keen, G., and Benazon, D. B. (1959b), *Lancet i*, 745.
(Other articles consulted: *British Medical Bulletin*, Vol. 17, No. 1, especially pp. 37–42; *Guy's Hospital Reports* Vol. 108, especially pp. 291–313.)

